# Transforming operating rooms into intensive care units and the versatility of the physician anesthesiologist during the COVID-19 crisis

**DOI:** 10.6061/clinics/2020/e2023

**Published:** 2020-06-08

**Authors:** Maria José Carvalho Carmona, Vinícius Caldeira Quintão, Brigite Feiner de Melo, Rodrigo Guerson André, Rafael Priante Kayano, Beatriz Perondi, Anna Miethke-Morais, Marcelo Cristiano Rocha, Luiz Marcelo Sá Malbouisson, José Otávio Costa Auler-Júnior

**Affiliations:** I Disciplina de Anestesiologia, Hospital das Clinicas HCFMUSP, Faculdade de Medicina, Universidade de Sao Paulo, Sao Paulo, SP, BR; II Diretoria Clinica, Hospital das Clinicas HCFMUSP, Faculdade de Medicina, Universidade de Sao Paulo, Sao Paulo, SP, BR; III Departamento de Cirurgia, Hospital das Clinicas HCFMUSP, Faculdade de Medicina, Universidade de Sao Paulo, Sao Paulo, SP, BR

With the prospect of an increasing number of COVID-19 cases and with the city of Sao Paulo being the epicenter of the COVID-19 pandemic in Brazil, Hospital das Clinicas of the Faculdade de Medicina of the Universidade de Sao Paulo (HCFMUSP) initiated an action plan to designate the largest of its eight institutes to the treatment of the disease. With the progressive increase in the number of attended cases, at the end of March 2020, the 900 beds of HCFMUSP Instituto Central (ICHC) were exclusively allocated to patients with COVID-19. A further 200 intensive care (ICU) beds were added in the following month, including those adapted inside operating rooms. The final plan accounted for more than 300 ICU beds and included 34 operating rooms in the four blocks of the main ICHC surgical theater that were adapted to attend to one to four patients each. This resulted in a complex of four ICUs with up to 76 beds. The rearrangement and specialized review of medical gas, electricity, air conditioning, and data network installations were essential procedures to ensure the safe use of these areas for this new purpose.

Following the cancellation, postponement, or transfer of surgical schedules at other institutes of the HCFMUSP complex and Hospital Universitario, the ICHC team of 117 physician anesthesiologists was divided into those remaining at the ICHC or conducting specific procedures at the destination sites, with emphasis on trauma, surgical emergencies, transplants, and high-risk obstetrics. According to the lessons learned from countries that have already gone through the peak of the pandemic, such as China and Italy, the 60 physician anesthesiologists who stayed in the ICHC were intensely retrained regarding the correct use of personal protective equipment and techniques of donning and doffing to minimize the occupational risk of COVID-19.

Physician anesthesiologists have previously been trained and are specialists in accessing the airway, including dealing with airway difficulty, and the transportation of critically ill patients. Retraining therefore focused on the safe tracheal intubation of COVID-19 patients, with respect to patient care while minimizing exposure to droplets and aerosols. In addition, anesthetic care for surgical and obstetric patients with COVID-19, intra-hospital transport of critically ill patients, and the role of the physician anesthesiologist as an intensivist were part of the team's training.

On March 30, 2020, the ICHC Anesthesiology team took over the role of the Rapid Response Team services and started working as hospitalists, replacing the Internal Medicine team that previously performed this function. This decision was based on the need to pay particular attention to airway approaches in COVID-19 patients who required tracheal intubation at admission, in wards, and in ICUs. Besides minimizing occupational exposure, technical skills decrease the risk of adverse events such as cardiac arrest during tracheal intubation in hypoxic patients. The responsibility of intra-hospital transport of intubated patients using vasoactive drugs was also assumed by the physician anesthesiologists’ team. This team also collaborated in the training of non-anesthesiologists in airway management. The usual number of beds in ICU-Anesthesiology increased from 17 to 22 after transformation of post-anesthetic recovery beds into ICU beds. Additionally, 24 beds in two new ICUs at ICHC, as well as the four ICUs created within the surgical theater, are currently the responsibility of the Anesthesiology Division. Intensive care specialists who did or did not belong to the Anesthesiology Division were recruited as day-to-day managers for each ICU.

Although the number of physician anesthesiologists was adequate for the several activities undertaken during the day shifts, the reallocation of working hours rendered the number of physicians insufficient for night and weekend shifts. This scenario led to extra-institutional professional recruitment, allowing physician anesthesiologists from private hospitals in Sao Paulo city to fill the vacant positions. These professionals were also enrolled in at least three more ICUs, with up to 12 beds each. The direct or indirect participation of physician anesthesiologists in ICUs containing at least 150 beds optimized the service capacity for severe cases of COVID-19 in the city of Sao Paulo.

With the increase in demand for mechanical ventilators and the market shortage of specific equipment for severe cases, the use of anesthesia machines as mechanical ventilators is necessary in ICUs. This has been successfully applied in other countries (1). The ICHC has a technologically modern set of anesthesia equipment, which provides adequate resources for prolonged respiratory assistance and weaning from mechanical ventilation. Together with the biomedical engineering team, several tests were carried out to adapt the anesthesia machines available in the surgical theater for use in ICUs. Other anesthesia machines that were placed at diagnostic and therapeutic sites or that were made available from other institutes of the HCFMUSP complex were also tested and adapted. When used in intensive care, the frequency of the need to exchange the soda lime, which is used to absorb carbon dioxide and reduce the consumption of gases and inhalation anesthetics, is reduced with the use of increased gas flow (2). The use of anesthesia machines as ICU ventilators also makes it possible to sedate patients using inhalation anesthetics. However, this procedure presents technical limitations when using the mechanical ventilators commonly used in ICUs (3).

Besides the implementation of new practices on the basis of the best evidence available in the literature, these medical care activities provide opportunities for specific clinical research in anesthesiology or in partnership with other disciplines. Furthermore, the clinical demand in these unprecedented times can encourage technological innovation and university-industry partnerships, especially with the Escola Politecnica of the Universidade de Sao Paulo and private companies. By following the appropriate protocols, this could promote the development of new materials and equipment that could improve the economic-industrial complex of healthcare in Brazil.

The significant participation of physician anesthesiologists during the COVID-19 crisis at HCFMUSP has corroborated the versatility of these specialists, as has already been demonstrated in other countries (1). Physician anesthesiologists, who are skilled team members, are collaborating in the informal training of newly hired nursing teams to care for critically ill patients and are showing physiotherapists how to use anesthesia machines as mechanical ventilators in ICUs. The environment is also one of great mutual learning. For instance, the experience of physician anesthesiologists and nurses in several types of surgery in the pronation position contributes to minimizing the risks related to the intubation and extubation of patients in ICUs. Another important benefit of this teamwork is the psychological support offered by the Psychology Division and the Psychiatric team of the Instituto de Psiquiatria of HCFMUSP (IPq) to physicians, residents, and other health professionals involved in the treatment of COVID-19 patients, including physician anesthesiologist staff and residents. As a fortunate consequence of this intense training and awareness program, very few anesthesiologists are getting infected within the hospital environment.

Changes and adaptations, in consideration of both patients and health care teams, are continuously being implemented. The anesthesiology field has incorporated various models from aviation checklists throughout the years, and in times of crisis, adopting strict protocols can contribute to improved results.
