# Erratum for: Transforming operating rooms into intensive care units and the versatility of the physician anesthesiologist during the COVID-19 crisis

**DOI:** 10.6061/clinics/2020/e2023err

**Published:** 2020-07-15

CLINICS 2020;75:e2023

In the article **Transforming operating rooms into intensive care units and the versatility of the physician anesthesiologist during the COVID-19 crisis**

**Replace** “Maria José Carvalho Carmonahttps://orcid.org/0000-0002-3031-2924,^*^ Vinícius Caldeira Quintãohttps://orcid.org/0000-0001-6459-7983, Brigite Feiner de Melo, Rodrigo Gherson André, Rafael Priante Kayano, Luis Marcelo Sá Malbouissonhttps://orcid.org/0000-0002-3261-5603, José Otávio Costa Auler-Júniorhttps://orcid.org/0000-0002-3919-1743” and “Disciplina de Anestesiologia, Hospital das Clinicas HCFMUSP, Faculdade de Medicina, Universidade de Sao Paulo, Sao Paulo, SP, BR” and “Carmona MJC, Quintão VC, Melo BF, André RG, Kayano RP, Malbouisson LM. Transforming operating rooms into intensive care units and the versatility of the physician anesthesiologist during the COVID-19 crisis. Clinics 2020;75:e2023” for:

**Replace** “Maria José Carvalho Carmonahttps://orcid.org/0000-0002-3031-2924^I^^*^ Vinícius Caldeira Quintãohttps://orcid.org/0000-0001-6459-7983,^I^ Brigite Feiner de Melo,^I^ Rodrigo Guerson André,^I^ Rafael Priante Kayano,^I^ Beatriz Perondi,^II^ Anna Miethke-Moraishttps://orcid.org/0000-0002-5077-4122,^II^ Marcelo Cristiano Rochahttps://orcid.org/0000-0001-6821-2286,^III^ Luis Marcelo Sá Malbouissonhttps://orcid.org/0000-0002-3261-5603,^I^ José Otávio Costa Auler-Júniorhttps://orcid.org/0000-0002-3919-1743^I^” and “^I^ Disciplina de Anestesiologia, Hospital das Clinicas HCFMUSP, Faculdade de Medicina, Universidade de Sao Paulo, Sao Paulo, SP, BR. ^II^ Diretoria Clinica, Hospital das Clinicas HCFMUSP, Faculdade de Medicina, Universidade de Sao Paulo, Sao Paulo, SP, BR. ^III^ Departamento de Cirurgia, Hospital das Clinicas HCFMUSP, Faculdade de Medicina, Universidade de Sao Paulo, Sao Paulo, SP, BR.” and “Carmona MJC, Quintão VC, Melo BF, André RG, Kayano RP, Perondi B, et al. Transforming operating rooms into intensive care units and the versatility of the physician anesthesiologist during the COVID-19 crisis. Clinics 2020;75:e2023”

